# Dissecting the tumor microenvironment of epigenetically driven gliomas: Opportunities for single-cell and spatial multiomics

**DOI:** 10.1093/noajnl/vdad101

**Published:** 2023-08-21

**Authors:** Jonathan H Sussman, Jason Xu, Nduka Amankulor, Kai Tan

**Affiliations:** Graduate Group in Genomics and Computational Biology, Perelman School of Medicine, University of Pennsylvania, Philadelphia, Pennsylvania, USA; Medical Scientist Training Program, University of Pennsylvania, Philadelphia, Pennsylvania, USA; Graduate Group in Genomics and Computational Biology, Perelman School of Medicine, University of Pennsylvania, Philadelphia, Pennsylvania, USA; Medical Scientist Training Program, University of Pennsylvania, Philadelphia, Pennsylvania, USA; Department of Neurosurgery, Perelman School of Medicine, Philadelphia, Pennsylvania, USA; Department of Pediatrics, Perelman School of Medicine, University of Pennsylvania, Philadelphia, Pennsylvania, USA; Center for Childhood Cancer Research, Children’s Hospital of Philadelphia, Philadelphia, Pennsylvania, USA

**Keywords:** histone-mutant glioma, IDH-mutant glioma, pediatric glioma, Single-cell sequencing, Spatial omics

## Abstract

Malignant gliomas are incurable brain neoplasms with dismal prognoses and near-universal fatality, with minimal therapeutic progress despite billions of dollars invested in research and clinical trials over the last 2 decades. Many glioma studies have utilized disparate histologic and genomic platforms to characterize the stunning genomic, transcriptomic, and immunologic heterogeneity found in gliomas. Single-cell and spatial omics technologies enable unprecedented characterization of heterogeneity in solid malignancies and provide a granular annotation of transcriptional, epigenetic, and microenvironmental states with limited resected tissue. Heterogeneity in gliomas may be defined, at the broadest levels, by tumors ostensibly driven by epigenetic alterations (IDH- and histone-mutant) versus non-epigenetic tumors (IDH-wild type). Epigenetically driven tumors are defined by remarkable transcriptional programs, immunologically distinct microenvironments, and incompletely understood topography (unique cellular neighborhoods and cell–cell interactions). Thus, these tumors are the ideal substrate for single-cell multiomic technologies to disentangle the complex intra-tumoral features, including differentiation trajectories, tumor-immune cell interactions, and chromatin dysregulation. The current review summarizes the applications of single-cell multiomics to existing datasets of epigenetically driven glioma. More importantly, we discuss future capabilities and applications of novel multiomic strategies to answer outstanding questions, enable the development of potent therapeutic strategies, and improve personalized diagnostics and treatment via digital pathology.

**Key Points**

Epigenetically driven gliomas harbor unique single-cell and spatial profiles;
IDH and histone mutations result in distinct microenvironment features;
Novel spatial omics technologies present a rich opportunity to study glioma subtypes

Cancers arising from glial cells (gliomas) are nearly universally fatal with limited therapeutic options aside from surgical resection, chemotherapy, and radiotherapy. Glioblastoma (GBM), the most aggressive form, has a 5-year survival of less than 5%.^1^ The advent of single-cell sequencing, and more recently, spatial omics technologies, have catalyzed the rate of discovery in brain tumors and represent a promising frontier to inform the development of novel therapies. Taken together, these “multiomic” technologies capture tens of thousands of transcriptional and epigenetic features within individual cells through various sequencing and imaging approaches, allowing for the vast spectrum of cell states and interactions to be studied at an unprecedented resolution.^2–4^ A growing volume of studies features these techniques along with increasing experimental sophistication and computational power in order to dissect and characterize both the heterogeneity and the common features in the glioma microenvironment. These studies have uncovered core principles of glioma pathogenesis^5–7^ which motivate promising novel therapeutic strategies for this devastating disease including the identification of actionable targets for differentiation therapy^8^ and immunotherapy^9^ among other approaches.

Many existing studies focus on isocitrate dehydrogenase (IDH)-wild type (WT) glioblastoma, the most common form of high-grade glioma, with many of the seminal studies and single-cell atlases specifically excluding cases with mutations in IDH or core histone proteins.^5,10,11^ Notably, the epigenomic features and unique microenvironmental landscapes (eg, the near-complete paucity of lymphocytes) suggest that high-dimensional profiling with multiomic technologies is aptly suited to study such tumors. IDH mutations define a subset of high-grade glioma and >80% of diffuse low-grade gliomas, of which 50-75% progress to become World Health Organization (WHO) grade IV tumors.^12^ Histone mutations (H3K27 and H3G34) are common in pediatric gliomas, with the H3K27M mutation found in 80% of diffuse intrinsic pontine gliomas,^13^ as well as in adult midline glioma. The WHO 2021 CNS update incorporates many additional genomic alterations and defines IDH-mutant (Mut) astrocytoma, IDH-Mut oligodendroglioma, and H3K27-altered diffuse midline glioma, among others, as separate entities from IDH-WT glioblastoma, recognizing their distinct clinical and histopathological features.^14^

IDH and histone mutations are mutually exclusive, though both create unique, non-stochastic global epigenetic dysregulation resulting in cellular reprogramming and oncogenesis. Activating mutations in IDH (most commonly IDH1-R132H) produce the oncometabolite 2-hydroxygluatarate (2-HG), which inhibits DNA and histone demethylases, among other oncogenic epigenomic changes such as PDGFRA activation.^12,15^ In neoplastic cells, this results in the characteristic G-CIMP signature defined by CpG island hypermethylation,^16^ and 2-HG has far-reaching effects on the tumor microenvironment such as its ability to suppress antitumor T cell activity.^17^ The most common histone mutation (H3K27M) results in epigenetic dysregulation via global H3K27me3 reduction through PRC2 inhibition among other mechanisms.^13^ This epigenetic dysregulation allows for the accumulation of potent oncogenic genomic alterations (*TP53*, *ATRX*, *PTEN*, *CDKN2A*, etc.) and malignant transformation. We collectively refer to these glioma subtypes (IDH/H3 mutant) as epigenetically driven gliomas.

The current review summarizes mechanistic insights gleaned from single-cell and spatial multiomic datasets and analyses of epigenetically driven gliomas (Table 1) and proposes future directions that may yield therapeutic vulnerabilities and clinical utility.

**Table 1.** Single-Cell Omics and Spatial Imaging Datasets of Epigenetically Driven Glioma

Reference	Cohort Size	Technology	# Cells	Cell types +/− Enrichment	Main Finding	Data Accessibility
Tirosh et al. (2016)^17^	*n* = 6 IDH1/2-Mut gliomas	scRNA-seq (SMARTseq2)	4347	Neoplastic cells	Identification of cancer stem cells in glioma	GSE70630
Venteicher et al. (2017)^18^	*n* = 10 IDH-A; *n* = 6 IDH-O gliomas	scRNA-seq (SMARTseq2)	14 226; 9879 (IDH-A); 4347 (IDH-O)	Neoplastic cells, TME	IDH-A and IDH-O share similar developmental hierarchy but vastly different TME	GSE40853
Müller et al. (2017)^42^	*n* = 5 GBM, *n* = 2 IDH-Mut glioma	scRNA-seq (Fluidigm C1 + 10x)	4181	Neoplastic cells, TAM enriched fraction	Glioma-specific tumor-associated macrophage signatures	EGAS00001002185; EGAS00001001900
Filbin et al. (2018)^26^	*n* = 6 H3K27M-gliomas	scRNA-seq (SMARTseq2)	2458	Neoplastic cells, microglia, and oligodendrocytes	H3K27M-glioma is composed of cells resembling oligodendrocyte precursors and is enriched for stem-cell-like profiles; identification of OPC-like marker PDGFRA as a novel target	GSE102130
Al-Ali et al. (2019)^21^	*n* = 5 IDH-Mut gliomas	scATAC-seq (Fluidigm)	336	Neoplastic cells	Pipeline development for integrated multimodal analysis	GSE137266
Wang et al. (2019)^19^	*n* = 22 IDH-WT GBM; *n* = 6 IDH-Mut gliomas	snRNA-seq (*n* = 19, 10x), scRNA-seq (*n* = 3, Fluidigm), scATAC-seq (*n* = 8)	31 281 (10X); 291 (Fluidigm);	Neoplastic cells, TME (non-enriched)	GBMs contain hierarchies of mesenchymal and proneural GSCs. IDH-Mut gliomas contain hierarchies of AC-like, OC-like, and stem-like states	EGAS00001002185, EGAS00001001900, and EGAS00001003845
Kampa et al. (2020)^57^	*n* = 25 GBM, n = 6 IDH-Mut gliomas	MALDI-MSI (spatial metabolomic profiling)	N/A	N/A; metabolomic analytes only	Tumor heterogeneity was readily seen on metabolite level; 2-HG accumulation detected in IDH-Mutated gliomas	NA
Chen et al. (2020)^33^	*n* = 16 H3G34R/V-gliomas (*n* = 6 PDGFRA-WT; *n* = 10 Mut); *n* = 3 Matched normal	scRNA-seq (10x)	64 943	Neoplastic cells; non-enriched TME	H3G34R/V mutant gliomas arise in GSX2/DLX+ interneuron progenitors which become blocked in differentiation and dependent on PDGFRA signaling	EGAS00001004301 (EGA)
Friebel et al. (2020)^40^	*n* = 22 Glioma; *N* = 14 brain met; *n* = 2 non-tumor	Mass Cytometry (74 immune-markers)	N/A	Lymphoid/myeloid cells	Microglia-derived phagocytes are abundant in the glioma TME vs. invading leukocytes in brain metastases	Mendeley (jk8c3c3nmz)
Babikir et al. (2021)^22^	*n* = 10 IDH-A; IDH-O	snATAC-seq (10x)	38 552	Neoplastic cells, glia, monocytes	Subtype-specific TF expression and targeting in IDH-Mut gliomas; ATRX as a key TF in IDH-Mut gliomas	GSE155430
Chaligne et al. (2021)^24^	*n* = 7 GBM, *n* = 7 IDH-Mut gliomas	scRNA-seq (SMART-seq2); scDNA-methylation (mscRRBS)	1786 (scRNA-seq); 1585 (scDNAme)	Neoplastic cells (CD45- enriched)	DNA methylation changes reflect glioma cellular states and contribute to propagation; differentiation is greater than de-differentiation in IDH-Mut glioma	GSE151506; GSE138794
Alghamri et al. (2021)^46^	*n* = 8 IDH-WT, *n* = 10 IDH-Mut gliomas	scRNA-seq (10x)	9765 (WT) 17 452 (Mut)	TME-derived granulocytes (granulocytes, macrophages, microglia, mono, and DCs); Neoplastic cells	Lower frequency of PMN-MDSCs in mice/human IDH-Mut gliomas may explain differential sensitivity to immunotherapeutics	GSE152277
Matthewson et al. (2021)^47^	*n* = 16 GBM; *n* = 15 IDH-Mut gliomas	5’ scRNA-seq/ SMART-seq2	8252 (SMARTseq2) 25 256 (10 × 5’)	CD3+ T-cells: CD4, CD8, (cycling, memory, effector, and regulatory)	Gene expression landscape of infiltrating T-cells in GBM; Nomination of new immunomodulatory targets (ie, CLEC2D/CD161)	GSE163108
Johnson et al. (2021)^25^	*n* = 6 IDH-Mut; *n* = 5 IDH-WT	scRNA-seq, scDNAme; bulk WGS/DNAme/RNA-seq	55 284 (scRNA-seq); 914 (scDNAme)	Neoplastic, TME	DNAme disorder is associated with early genetic lesions, altered cell states, and increased cell plasticity	EGAS00001005300
Friedrich et al. (2021)^45^	*n* = 5 IDH-WT, *n* = 5 IDH-Mut; *n* = 7 Normal control	scRNA-seq (*n* = 17) bulk-RNA-seq (*n* = 14)	4460	Microglia and Macrophage (CD45+ CD3-CD19-CD20- enriched)	Identification of glioma-associated macrophage/microglia cell states in IDH-Mut gliomas; 2-HG regulation of tryptophan catabolism in myeloid cells.	GSE166420
Yin et al. (2022)^43^	*n* = 4 IDH-WT GBM; *n *= 3 IDH-A gliomas	snRNA-seq (10x)	24 227	Monocytes and microglia	Higher proportion of monocytes is associated with worse outcomes in GBM	PRJCA008116 (National Genomics Data Center of China)
Jessa et al. (2022)^31^	*n *= 43 HGG and PFA-ependymomas; *n* = 24 H3K27M; *n *= 5 PFA-EP	scRNA-seq (24), scATAC-seq (4), 10x Multiome (10)	181 282	Neoplastic, immune cells, and vascular	K27M mutations originate in distinct lineages with defined positional identities	EGAS00001005773
Gulaia et al. (2022)^35^	*n* = 3 IDH/p53-Mut gliomas; *n* = 3 IDH/p53-wt gliomas	Full-length snRNA-seq	576	Neoplastic cells (enriched)	Novel targets and aberrant pathways of IDH/TP53-Mut glioma stem cells	GSE164624
Liu et al. (2022)^28^	*n* = 50 H3K27M-diffuse midline gliomas	scRNA-seq (*n* = 18); snRNA-seq (*n* = 25); snATAC-seq (*n* = 8); in situ sequencing (HybISS, *n* = 14 × 116 markers); CODEX (*n* = 4 × 19 markers)	N/A	Neoplastic cells, TME, and oligodendrocytes	Universal presence of OPC-like cells across spectrum of ages and anatomical presentations, identification of mesenchymal cells in H3K27M-gliomas	GSE184357 (scRNA/snATAC); Zenodo (6805729, HybISS)
Karimi et al. (2023)^52^	*n* = 139 HGG (19 IDH-Mut); *n* = 46 brain mets	imaging mass cytometry	1 163 362	Neoplastic cells, astrocytes, and TME	Correlation of multicellular structures in brain TME with outcome	Zenodo (7383719)
Ren et al. (2023)^27^	*n* = 5 IDH-WT GBM, *n* = 2 IDH-Mut GBM, *n* = 5 H3K27M DMG	Spatial transcriptomics (10× Visium), Oxford Nanopore	26 460 (spots)	Neoplastic cells, TME, and radial glia	Identification of tumor niche-specific regulatory patterns of radial glial stem-like cells	HRA001865 (short read) HRA001960 (long read)

## IDH-Mutant Tumor Cell States

### Single-Cell RNA-Sequencing-Based Transcriptional States

Gliomas are fundamentally characterized by deranged cellular differentiation, with cell states that recapitulate the trajectory of the developing brain.^6^ Verhaak et al. were the first to describe a trancriptomically defined GBM classification scheme based on bulk RNA-sequencing, yielding four unique molecular subtypes: Proneural, neural, classical, and mesenchymal.^18^ Notably, IDH-mutant (Mut) tumors displayed exclusively proneural or neural signatures. More recently, single-cell sequencing has demonstrated that individual neoplastic cells fall along a spectrum of differentiation. Profiling of 6 IDH-Mut oligodendrogliomas by Tirosh et al. identified a cellular hierarchy in which proliferating stem-like cells give rise to astrocytic (AC) and oligodendrocytic (OC) tumor cells. This hierarchy was shown to be independent of tumor subclonality, such that derivatives of a single progenitor cell stochastically traverse either an AC or OC lineage.^19^

This work was expanded by Venteicher et al. with a larger cohort of 16 patients across IDH-Mut astrocytomas (IDH-A, characterized by secondary *TP53* and *ATRX* mutations) and IDH-Mut oligodendrogliomas (IDH-O, with codeletion of chromosome arms 1p and 19q).^20^ This study discovered multiple salient findings that were shared across IDH-A and IDH-O gliomas. For example, intra-subtype cellular lineage trajectories displayed nearly fixed ratios, and higher-grade tumors exhibited a greater proportion of cycling/undifferentiated cells. IDH-WT tumors differed markedly in their lineage composition, with a shift towards the AC-like lineage. Interestingly, whereas AC-like and OC-like lineage states are inversely correlated and mutually repressive in IDH-O tumors, IDH-A tumors feature multiple lineage identities within individual cells, suggesting that IDH-A represents an intermediate transcriptional state between IDH-O and IDH-WT glioma.

Wang et al. similarly identified three clusters of tumor cells across IDH-Mut gliomas, termed IDH-A, IDH-O, and IDH-S (stem-like) cells.^21^ Subsequent work by Neftel et al. expanded these observations for IDH-WT glioma, identifying four core tumor cell states (astrocyte-like, oligodendrocyte progenitor (OPC)-like, neural progenitor (NPC)-like, and mesenchymal), and experimentally demonstrated plasticity between these states using a lineage-tracing system.^5^ While the proneural to mesenchymal axis primarily captures the spectrum of neoplastic cell states in IDH-WT glioma,^21,22^ a distinctly mesenchymal state has not yet been described in de novo IDH-Mut tumors. However, integrated atlases of multiple datasets can greatly increase the power to uncover novel cell phenotypes^10^ and are much needed to corroborate the idea of transcriptional plasticity in astrocytic IDH-Mut gliomas. Importantly, a large study of 86 primary-recurrent patient-matched paired samples of IDH-WT GBM revealed a shift from proneural towards mesenchymal phenotypes using scRNA-seq.^22^ Through single-cell deconvolution of bulk RNA-seq, an analysis of 304 longitudinal patients demonstrated that IDH-Mut gliomas exhibit distinct cell-type changes during progression, tending to maintain a proneural phenotype and displaying an increase in stem-like neoplastic cells and a decrease in differentiated-like neoplastic cells.^23^ However, an analogous study at the single-cell level has not yet been undertaken.

Differentiation states alone may not adequately capture important (and potentially targetable) cell populations or explain physiologic activity in glioma cells. One report in IDH-WT glioma proposed an scRNA-seq-based classification scheme of 4 states based on both differentiation and metabolic pathway expression: Glycolytic/plurimetabolic, mitochondrial, neuronal, and proliferative/progenitor.^24^ Another report with scRNA-seq of 2 IHD-Mut gliomas and 16 IDH-WT gliomas presented tumor cells stratified by: Epithelial-to-mesenchymal transition, MYC-targets, interferon-gamma response, and hypoxia signatures.^9^ In IDH-Mut glioma, sequencing of 576 SOX2+ sorted cells (putative glioma stem cells) across 6 patients uncovered rare populations with differential metabolic properties that are independent of underlying differentiation states.^25^

Lastly, following the seminal work by Venkatesh et al. demonstrating that glioma cell synaptic integration promotes tumor progression,^26^ a recent analysis of an integrated scRNA-seq dataset of 4 IDH-WT and 3 IDH-Mut tumors suggests that IDH-Mut tumors harbor unique GABAergic neuron-like neoplastic cells which are marked by SOX11.^27^ Functional validation using a patch-clamp demonstrated that these cells uniquely exhibited neurophysiological properties. Taken together, these findings suggest that IDH-Mut tumors possess distinct metabolic and electrophysiologic properties that correspond to unique clinical and pathological features.

### Chromatin-Based and Methylomic Cell State Interrogation of IDH-Mut Gliomas

An initial single-nucleus assay for transposase accessible chromatin using sequencing (snATAC-seq) study of IDH-Mut gliomas consisting of only 336 cells corroborated stratification of IDH-Mut gliomas by the IDH-A/O classification.^28^ However, a later study of 22 IDH-A/O gliomas (>38 000 cells, Babikir et al.) recapitulated the prior three scRNA-seq-based cell states (AC-like, OC-like, and neural stem cellneural stem cell (NSC)-like).^29^ Remarkably, this study identified increased open chromatin epigenomic states in IDH-A tumors, and linked astrocytic/oligodendrocytic phenotypes to transcription factor induction by secondary mutations (*ATRX*, 1p/19q-codeletion). A recent single-cell multiomic analysis by Wei et al. suggested that both IDH-A and IDH-O gliomas resemble early stages of the oligodendrocyte lineage with a differentiation block due to hypermethylation and suppression of essential regulators of the myelination program.^30^ This analysis further elucidates the fundamental mechanistic role of IDH mutations across IDH-Mut astrocytomas and oligodendrogliomas and motivates a potential differentiation therapy approach for IDH-Mut glioma. Importantly, these studies emphasize the need for multiomic interrogation and epigenetic profiling of IDH-Mut glioma.

Lastly, novel methods of single-cell DNA methylation profiling are able to capture the dysregulated DNA methylation unique to IDH-Mut gliomas. Chaligne et al. applied multimodal single-cell reduced-representation bisulfite methylation sequencing (RRBS) + scRNA-seq + targeted genotyping in ~800 IDH-Mut and ~800 IDH-WT cells across 14 patients.^31^ They identified clear separation of methylation patterns between IDH-Mut and IDH-WT tumors and identified more granular localization of epigenomic dysregulation, including unique enhancer hypermethylation with differentiation to AC and OC-like cells and decoupling of DNA methylation and expression at distinct genomic loci related to expression of cell cycle and proliferation genes. A separate multiomic study of ~1000 single-cell DNA methylomes and concomitant scRNA-seq similarly found that methylation patterns stratify IDH-Mut and IDH-WT cells, while superimposition of methylation and transcriptomic patterns identified unique cell states representative of distinct physiologic events, namely the cellular response to hypoxia.^32^

### Histone-Mutant Tumor Cell States

The characterization of histone-mutated gliomas has especially benefited from multiomic analyses. H3K27M-mutant gliomas were initially profiled at the single-cell level by Filbin et al., who identified a similar trajectory to that of IDH-Mut glioma involving proliferating stem cell-like cells which self-renew and differentiate into AC-like and OC-like cells (*n* = 6 patients).^33^ Notably, these studies inferred that the cell of origin/progenitor state in H3K27M-Mut glioma more closely resembled oligodendrocyte progenitor cells (OPCs), while IDH-Mut glioma progenitors resembled neural progenitor cells. Comparison of H3K27M-Mut glioma and IDH-Mut glioma reveal a higher fraction of non-proliferating OC-like cells in H3K27M-Mut glioma.^34^ These findings strongly imply that H3K27M histone mutations specifically induce a differentiation block along the OPC-OC axis and maintain a source of OPC-like progenitors to repopulate the tumor.^34^ Importantly, this work ascribes a unique functional role of OPCs to the maintenance of H3K27M-Mut gliomas.

In an expanded cohort of 50 pediatric gliomas, Filbin and colleagues assessed the relationship between anatomic and topographic features of histone-mutated glioma with distinct transcriptional/chromatin signatures.^35^ Remarkably, they identified distinct OPC-like progenitor populations specifically associated with tumor location along the dorso-ventral brainstem axis. Moreover, they noted an increasing mesenchymal transcriptional signature with age, being the first to identify a mesenchymal cell state in histone-mutant glioma. Additional epigenetic techniques are needed to expand the identification of therapeutically targetable cell states, with early results from epigenetic CyTOF on H3K27M-Mut glioma cell lines demonstrating significant future potential.^36^

Beyond differentiation states, identifying the exact cell of origin in glial malignancies is critical to identifying targetable pathways as well as for developing accurate in vitro and in vivo models. As such, multiomic single-cell profiling is necessary to answer this question. Interestingly, single-cell RNA and ATAC profiling of H3K27M-Mut gliomas stratified by whether the mutation occurs on the canonical H3.1/H3.2 histone variants or the noncanonical H3.3 variant suggested different cells of origin along the dorso-ventral axis,^37^ crucially advancing our understanding of the early phases of H3K27M-Mut glioma pathogenesis.

Unlike midline H3K27M-Mut gliomas, H3G34R/V-Mut gliomas are hemispheric and primarily affect adolescent patients.^38^ Single-cell RNA-seq of 16 H3G34R/V patients^39^ by Chen et al. revealed a transcriptional signature comprised mainly of neuronal and astrocyte-like cells with an absence of oligodendroglial-like cells. Most cells displayed an abnormal co-expression of interneuron and astrocytic gene signatures, suggesting significant divergence of lineage hierarchies in midline versus hemispheric histone-mutant glioma. They hypothesized that these tumors originate from GSX2+ interneuron progenitors with a differentiation block, and demonstrated that while the histone mutation is necessary to initiate oncogenesis, it is ultimately dispensable at later stages in which aberrant expression of PDGFRA is sufficient to maintain tumor viability and growth. This is in contrast with H3K27M-Mut gliomas, whereby the oncohistone is the core driver of the tumor at all stages. Overall, this suggests that the H3G34R/V mutations produce unique cell states driven by their neural cell of origin.

### Immune Microenvironment Characterization of Epigenetically Driven Gliomas

Single-cell omics approaches have emerged as a crucial tool for dissecting the tumor-immune microenvironment (TIME). It has been well established that the TIME of epigenetically driven gliomas differs from that of IDH-WT gliomas in key ways. Notably, IDH-Mut tumors exhibit immune-cold signatures characterized by a general paucity of immune cells.^40^ Furthermore, IDH-mut gliomas exhibit greater myeloid-to-lymphoid cell ratios, ostensibly predominated by microglial transcriptional signatures.^41,42^ Perhaps non-coincidentally, histone-mutant tumors are also largely immune cold.^43^ IDH-A tumors contain more myeloid cells (microglia and macrophages) than IDH-O tumors,^20^ and are enriched for monocytic-lineage cells from circulation. Interestingly, myeloid cells in IDH-O tumors are mostly comprised of microglia.^29^ In H3K27M-Mut gliomas, microglial transcriptional signatures are observed in pediatric patients, while adult patients exhibit more macrophage signatures.^33^ In all cases, a spectrum of macrophage to microglia phenotypes is observed, indicating that a binary classification of bone-marrow-derived macrophages and resident microglia as previously defined^44^ may not be sufficient to characterize these cells. However, considering that immune populations in gliomas are rare in general (compared with other malignancies) and especially depleted in epigenetically driven gliomas compared to IDH-WT gliomas, single-cell profiling of bulk tumors is generally only able to profile enough cells for a broad characterization.

Sorting of CD45+ cells followed by scRNA-seq enables finer dissection of immune cell types, and recent reports demonstrate that the IDH-Mut TIME is distinct from that of IDH-WT glioma. First, profiling of enriched myeloid cells across 4 IDH-WT and 3 IDH-Mut grade IV gliomas identified a predominance of TREM2+ macrophages in IDH-Mut glioma along with fewer monocytes and non-inflammatory macrophages.^45^ Recently, the largest profiling study to date by Gupta et al. characterized the immune cells of 18 gliomas across IDH mutation and recurrence status, and identified a unique subset of metabolically enriched microglia that were restricted to IDH-Mut gliomas.^46^ Moreover, they similarly identified a population of TREM2+ myeloid cells and showed in a mouse model that these cells mediate antitumor immunity in glioma, in contrast to the immunosuppressive role of TREM2 reported in other cancers.^46^ Perhaps more than for other cellular components of glioma, it is evident that single-cell multiomic analyses are desperately needed for lucid evaluation of the immune compartment.

In contrast to studies suggesting predominantly unidirectional roles for IDH-Mut myeloid cells, additional studies have revealed distinct immunosuppressive and inflammatory interactions between myeloid cells and tumor cells in IDH-Mut tumors. By profiling 8 IDH-WT/6 IDH-Mut tumors along with in vitro and in vivo studies, Friedrich et al. directly implicated the oncometabolite 2-hydroxyglutarate in modulating macrophage tryptophan metabolism, resulting in increased immunosuppression in IDH-Mut tumors. They subsequently demonstrated that targeting tryptophan metabolism can reverse this immunosuppression.^47^ In a second study of immune cell sequencing of both mouse and human glioma (*n* = 8 IDH-WT, 10 IDH-Mut), Alghamri et al. identified that G-CSF secreted by stem cell-like cells in IDH-Mut glioma (detectable in human serum samples) induces proliferation of non-immunosuppressive granulocytes, and demonstrated the therapeutic potential of G-CSF in a mouse glioma model.^48^ While IDH-Mut glioma is characterized by an immunosuppressive microenvironment, multiomic analyses have the potential to uncover targetable interactions for immunomodulatory therapeutics.

In addition to myeloid cells, a glioma T cell atlas from 16 IDH-WT and 15 IDH-Mut gliomas by Mathewson et al. demonstrates important differences in the lymphoid populations. Notably, while both tumor types shared a similar composition of infiltrating T cell subsets, both CD4+ and CD8+ T cell populations were greater in number and had greater cytotoxicity, interferon, and cellular stress programs in IDH-WT glioma.^49^ Importantly, their key finding that the CD161 receptor inhibits T cell-mediated killing of glioma is relevant independent of IDH status.

Overall, single-cell studies have revealed that IDH-Mut glioma harbors a complex TIME with both immunosuppressive and immune activating factors that drive the clinical course of the disease and may yield therapeutic opportunities. Notably, there is a paucity of single-cell immune data for histone-mutant gliomas, with many open questions as to the mechanisms of immune evasion in those glioma subtypes.

### Cellular Interactions and Spatial Analyses

Spatial profiling technologies are still in their infancy, but present unprecedented opportunities to study the tumor microenvironment in gliomas. Paired with robust computational approaches, these techniques can identify ligand-receptor interactions, characterize specialized cellular neighborhoods, and uncover spatially defined biomarkers. Current work is limited thus far, especially with regard to epigenetically driven tumors. However, recent work in IDH-WT tumors has demonstrated the utility of these approaches, despite limited sample sizes. For example, spatial proteomics using co-detection by indexing (CODEX)^50^ revealed localized shifts in immune cell composition in GBM explants under immunotherapy,^51^ low-plex RNA-FISH directly visualized paracrine signaling between neoplastic cells and adjacent nonmalignant glia,^22^ and spatial RNA-sequencing identified spatially distinct transcriptional programs and an immunosuppressive tumor-myeloid niche.^52^ Spatial proteomics enables direct spatial immunophenotyping, correlation with protein stains used in routine neuropathology, quantification of extracellular features, and specific identification of neoplastic cells through the detection of mutant IDH or histone proteins when applicable. However, these approaches can only quantify up to around 60 targets. Spatial transcriptomic approaches allow for the precise quantification of hundreds to thousands of RNA targets, yet are less equipped to study extracellular features and are less translationally applicable.

In the largest spatial profiling effort to date, Karimi et al. applied imaging mass cytometry, which employs metal-tagged antibodies followed by precise laser ablation and quantification. They measured 42 protein targets, profiling 389 images across 139 HGGs and 46 brain metastases, including 19 images from IDH-Mut glioma.^53^ The analysis of IDH-Mut tumors was limited to recapitulating key findings about immune infiltration, notably that NK cells and macrophages are reduced in IDH-Mut tumors. These limitations are primarily due to the use of a broad antibody panel with limited resolution to discern tumor cell states. Current spatial proteomic analysis of histone-mutant tumors is quite minimal, with published datasets limited to a 24-plex cyclic immunofluorescence panel (CyCIF)^54^ on 11 H3K27M-Mut tissue microarray cores^54^ and an 8-plex CODEX panel in 4 H3K27M-Mut gliomas.^35^

Spatial transcriptomics can be accomplished via hybridization of fluorescent oligonucleotide probes with direct visualization, or via RNA-capture and next-generation sequencing of regions (“spots”) on a microfluids platform. The former allows for high resolution and high accuracy at the single-cell level but requires a customized gene panel limited to several hundred targets. The latter allows for sequencing of the whole transcriptome, but is currently limited to a resolution of 1 to 10 cells per spot and thus requires computational deconvolution approaches, although it will likely achieve true single-cell resolution in the near future. There is a growing range of methods including MERFISH,^55^ seqFISH,^56^ GeoMx,^57^ Slide-seq,^58^ and the 10x Genomics platforms, Visium and Xenium. The largest spatial transcriptomics dataset for IDH-Mut tumors was generated by Kuchroo et al. using the 10x Genomics Visium platform and includes 10 IDH-Mut astrocytomas across WHO grades II–IV.^59^ Through a novel ligand-receptor simulation analysis, they enforced the significance of the previously established SPP1-CD44 signaling axis in glioma and identified that genes related to hypoxia regulation and antigen presentation are directly associated with this ligand-receptor interaction.^59^

As described earlier, Liu et al. complemented their single-cell RNA and ATAC atlas of H3K27M-mutant gliomas with spatial transcriptomics via hybridization-based in situ sequencing (HybISS),^60^ which utilizes the amplification of barcoded probes, on 16 regions across 14 patients with a 116 cell-type-specific gene panel.^35^ Interestingly, they found that AC-like cells constitute the major malignant cell compartment when assessed via HybISS, challenging the consistent finding by scRNA-seq that OPC-like stem cells are the major cell type in H3K27M-Mut glioma. The authors hypothesize that cell dissociation during sequencing protocols (which is not required in spatial imaging) could differentially damage AC-like cells, enforcing the need for orthogonal single-cell profiling techniques. Notably, this observation does not challenge the key hypothesis that there is a differentiation block to the OC-like state. In addition, the authors identified a pattern of colocalization between vascular cells and mesenchymal cells as well as a niche of cycling OPC-like and OC-like cells surrounded by non-proliferating AC-like cells.

A single study thus far, by Ren et al., integrates spatial profiling for IDH-WT (*n* = 3), IDH-Mut (*n* = 2), and H3K27M-Mut (*n* = 5) glioma.^61^ They applied 10x Genomics Visium spatial transcriptomics and identified four spatially defined gene expression modules conserved across samples representing hypoxic, vascular, invasive, and tumor core niches. They found that OPCs were most abundant in the tumor core but were present in all niches. Importantly, they found that radial glia-like cells were most enriched in the neuron-rich invasive niche in H3K27M-Mut and IDH-WT samples but in the IDH-Mut samples, these cells were most enriched in the hypoxic niche. They also found that invasive growth of these radial glia is stimulated by FAM20C within the invasive niche.^61^

Lastly, although less frequently implemented, spatial metabolomics via mass spectrometry holds the potential to identify metabolic niches with unique vulnerabilities, as demonstrated in IDH-WT glioma, where hypoxic niches were associated with increased genomic instability.^52^ This technique was applied to IDH-Mut gliomas by Kampa et al. with a cohort of 25 IDH-WT and 6 IDH-Mut gliomas, suggesting that IDH-Mut gliomas possess more perturbations in stromal metabolism compared to IDH-WT gliomas.^62^

### New Avenues for Treatment

Understanding the intricate complexities of tumor cell states and their interactions with immune and stromal components is crucial to developing desperately needed treatments. The integration of multiomic analyses across a range of modalities is an indispensable tool for the identification of therapeutic targets directed at both tumor-intrinsic and tumor-extrinsic (microenvironment) compartments of epigenetic gliomas. For example, pushing the cells towards a particular state could unlock a specific vulnerability which could be exploited via a combination therapy, a strategy that has been proposed as “differentiation therapy.”^63,64^ In the case of IDH-Mut and H3K27M-Mut gliomas, this would involve targeting the putative glioma stem cells (GSCs) that underlie tumor progression and resistance to therapy. In accordance with this principle, Panditharatna et al. identified via a CRISPR screen that OPC-like GSCs specifically maintain H3K27M-Mut glioma via the SMARCA4 gene regulatory network and the BRG1-BAF complex.^65^ SMARCA4 or BRG1-directed therapy depleted the OPC-like cell population, increased AC-like differentiation, and improved survival in a mouse model.

Single-cell approaches also provide strong insight into the effects of tumor therapy, which can identify biomarkers of therapy response and elucidate mechanisms of disease progression. While IDH-WT tumors have been shown to undergo a shift to the mesenchymal state under standard therapy,^22^ principles of tumor progression have not been fully characterized in epigenetically driven subtypes at the single-cell level. A report of patients with IDH-Mut oligodendroglioma under therapy with the mutant-IDH inhibitor ivosidenib (*n* = 7 untreated, 3 treated including 1 matched-pair) revealed a shift towards differentiated AC-like states and away from undifferentiated and proliferating cells through scRNA-seq profiling.^66^ Given the current clinical trials inhibiting other epigenetic regulators such as EZH2 and HDAC in gliomas,^63^ single-cell characterization of subsequent studies would greatly improve our understanding of epigenetic dysregulation and glioma cell differentiation. For example, through the computational inference of tumor subclones via novel methods which infer copy number variations in single-cell multiomics data,^67^ one can identify subclones that are evasive or resistant to therapy in order to predict treatment response and identify targeted combinatorial therapeutic strategies.

Finally, single-cell profiling of immune cells under therapy would elucidate processes of local and systemic immune modulation and provide insight into how to improve the efficacy of immunotherapies. Profiling of myeloid cells from the CSF of four patients undergoing GD2-CAR T cell therapy for H3K27M-Mut glioma, a promising early trial, revealed populations characterized by differential interferon response over time.^68^ As described earlier,^47^ tumor-associated myeloid cells are a promising target for therapeutic intervention, and additional profiling will elucidate how these cells respond to immunotherapy in clinical trial contexts.

## Future Directions and Opportunities

### Expanding Technologies and Outstanding Questions

**Figure 1.**
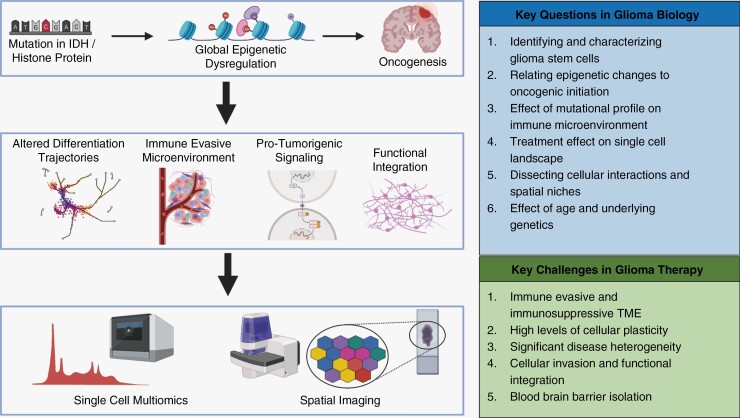
A single-cell approach to biomedical discovery for epigenetically driven gliomas. Point mutations in isocitrate dehydrogenase (IDH) or histone proteins create global epigenetic dysregulation, leading to alterations in transcription and metabolism resulting in oncogenesis. These subtypes of glioma lead to alterations in tumor-intrinsic and tumor-extrinsic features that are distinct from IDH-wild-type glioma. Addressing the key challenges in glioma therapy requires an understanding of how epigenetic dysregulation in these subtypes yields their unique features. Experimental and computational advances in single-cell and spatial multiomics provide the capacity to directly interrogate cellular interactions and spatial organization and address the key questions and challenges in glioma biology at unprecedented cellular resolution.

Single-cell and spatial profiling have revealed fundamental pathological processes that define cell-intrinsic oncogenic mechanisms and microenvironment dysregulation in epigenetically driven gliomas (Figure 1). The rapidly expanding repertoire of multimodal technologies is critical for further investigation of these glioma subtypes and for development of novel therapies. Paired multiomic profiling of ATAC and RNA will allow for a more precise association of epigenetic and transcriptional features by capturing both features within the same cell. This capability will likely prove to be critical in studying glioma as there may be a temporal lag between epigenetic and transcriptional changes. Therefore, this technology can more accurately dissect the developmental trajectory of glioma cells and capture novel therapeutic targets. Additionally, paired multiomic profiling of DNA and RNA can improve the identification of tumor subclones, which can be used to identify specific mechanisms of treatment resistance.^2^ The promising technologies of spatial ATAC^69^ and spatial CUT&Tag^70^ will vastly improve our understanding of the interplay between the tumor microenvironment and epigenetic reprogramming. These approaches utilize a microfluidics system to capture a range of epigenetic features at a resolution of up to 20 μm. In addition, new spatial transcriptomic technologies such as MERFISH, GeoMx, and Xenium can profile up to thousands of genes at subcellular resolution in brain tissue.^55^ MERFISH has been applied with a limited panel in IDH-WT glioma,^64^ but most of these technologies have not yet been utilized to study epigenetically driven gliomas.

Along with the addition of new technologies, single-cell multiomic approaches are needed to address a range of unanswered questions. More pediatric glioma atlases, such as the one developed by the Human Tumor Atlas Network,^71^ are needed to understand the influence of age on glioma pathogenesis and whether there are other key transcriptional and epigenetic programs unique to pediatric IDH-WT glioma that have yet to be identified. It is likely that additional epigenetic or multifactorial drivers exist in pediatric glioma that are distinct from adult tumors. Moreover, larger cohorts are also needed for a deeper understanding of these tumors in general, including for adult gliomas. Namely, the role of secondary mutations in epigenetically driven glioma has only been superficially defined,^29^ with an unclear role of key oncogenic driver genes such as *EGFR, PTEN*, *RB1*, *NF1*, *TERT*, and *CDKN2A* on modulating the tumor microenvironment. In addition, recent evidence suggests IDH-Mut gliomas induce systemic effects on the immune system.^72^ These effects likely mediate immune suppression and may even have the potential to serve as biomarkers to predict the molecular subtype of the tumor, yet peripheral effects have not been characterized at the single-cell level and represent an important future direction.

As single-cell omics techniques expand, relevant datasets of normal brain and sophisticated computational techniques are required to reconcile data gleaned from such strategies. To this end, the rapidly expanding single-cell omics datasets of non-neoplastic brain generated by consortium projects such as the Human Cell Atlas,^73^ Human Biomolecular Atlas Program (HuBMAP),^74^ and the BRAIN Initiative^75^ provide an invaluable resource for studying epigenetically driven gliomas. Reference mapping of non-tumor data onto glioma datasets is an important analytical tool for understanding cellular phenotypes and developmental trajectories. Therefore, larger datasets with more extensive profiling of rare cell types will be critical in dissecting the phenotypes of progenitor cells in epigenetically driven gliomas. Equally important is the computational ability to integrate datasets across modalities. Paired multimodal sequencing ameliorates this need in some cases, yet improved computational approaches will enable the expanded analysis of the rich compendium of existing datasets presented in this Review. Lastly, while there is a growing range of sequencing-based integration methods, there is an exciting opportunity for multiomic integration across sequencing and imaging modalities.^76,77^

### Multiomics in the Clinic

Currently, most high-dimensional multiomic profiling techniques discussed in the present Review are costly and require lengthy protocols and sophisticated laboratory techniques to perform successfully. However, there is a burgeoning opportunity for high-throughput spatial and multiomic technologies to provide direct clinical benefit in the context of digital pathology. For example, the multiplexed immunohistochemistry (IHC) InSituPlex system by Ultivue enables the simultaneous detection of up to 12 protein targets on a single tissue slide with high-throughput capacity on the order of only several hours of imaging time per slide.^78^ Alternatively, the COMET system by Lunaphore has demonstrated automated imaging of up to 40 markers in under a day.^79^ High-throughput approaches will not only optimize standard neuropathology workflows, but allow for the quantitation of higher-dimensional biomarkers which can have prognostic value and inform targeted treatment options. Namely, tissue cellular neighborhoods, which are recurrent functional patterns of spatial organization between cell types, have been shown to be useful variables for identifying and delineating tumor subtypes in other cancer types.^80,81^ Moreover, while there is an expanding toolkit of computational approaches to deconvolute spatial motifs and identify important patterns of cellular interaction,^82–84^ there remains a pressing need for analytical strategies to translate this data into clinically actionable information. In addition to applying multiplexed imaging directly, another avenue of clinical translation involves correlating spatial niches and topological features identified through spatial multiomics with histopathological features on a hematoxylin and eosin-stained slide. Given well-annotated training data, sophisticated machine learning algorithms are well suited to identify useful features that are not readily apparent to the eye of a pathologist and provide meaningful interpretations, and are well poised to greatly expand the scope of clinically relevant information garnered by routine neuropathology.

Single-cell sequencing technologies remain further away from direct clinical application. While they can be used to study the cellular response to therapy as previously noted, whole transcriptome and whole-genome approaches remain too complex and costly to provide routine diagnostic and therapeutic guidance. However, there is strong potential for their future utility in epigenetically driven glioma. For instance, single-cell sequencing may be necessary to identify therapeutic vulnerabilities that are present in rare stem cell-like populations that cannot be detected via deconvolution of bulk RNA-seq. In addition, key discoveries gleaned from multiomic profiling of transcriptional and epigenetic features may motivate the incorporation of bulk epigenetic profiling (ATAC-seq, ChIP-seq, etc.) into clinical practice. Lastly, while earlier in development, targeted single-cell sequencing approaches, such as those offered by 10x Genomics and BD Rhapsody are more applicable to clinical translation. By capturing a limited panel of genes, these methods greatly reduce cost and increase scalability while preserving the information most critical for clinical diagnostics and treatment planning. In conclusion, epigenetically driven gliomas present an exciting opportunity for high-dimensional multiomic data to uncover complex mechanisms and develop targeted treatments and will undoubtedly provide invaluable data as we seek meaningful life-extending therapies for these horrific brain tumors.
